# Editorial: Lipids and inflammation in health and disease, volume II

**DOI:** 10.3389/fcvm.2023.1174902

**Published:** 2023-04-12

**Authors:** Evgeny Bezsonov, Mirza S. Baig, Michael Bukrinsky, Veronika Myasoedova, Alessio Ravani, Vasily Sukhorukov, Dongwei Zhang, Victoria Khotina, Alexander Orekhov

**Affiliations:** ^1^ Laboratory of Angiopathology, Institute of General Pathology and Pathophysiology, Moscow, Russia; ^2^ Laboratory of Cellular and Molecular Pathology of the Cardiovascular System, Avtsyn Research Institute of Human Morphology, Petrovsky National Research Centre of Surgery, Moscow, Russia; ^3^ Department of Biology and General Genetics, I.M. Sechenov First Moscow State Medical University (Sechenov University), Moscow, Russia; ^4^ The Cell Physiology and Pathology Laboratory, Orel State University Named After I.S.Turgenev, Orel, Russia; ^5^ Department of Biosciences and Biomedical Engineering (BSBE), Indian Institute of Technology Indore (IITI), Simrol, India; ^6^ Department of Microbiology, Immunology and Tropical Medicine, School of Medicine and Health Sciences, The George Washington University, Washington, DC, United States; ^7^ Monzino Cardiology Center (IRCCS), Milan, Italy; ^8^ Diabetes Research Center, Beijing University of Chinese Medicine, Beijing, China; ^9^ Institute for Atherosclerosis Research, Skolkovo Innovative Center, Moscow, Russia

**Keywords:** lipids, inflammation, atherosclerosis, LDL, atherogenicity

**Editorial on the Research Topic** Lipids and inflammation in health and disease, volume II

The focus of this Research Topic is a continuation of the previous one and is devoted to the study of the role of lipids in inflammation in health and disease. Atherosclerosis is a classic example of how lipids involved in the normal functioning of cells and tissues can also induce inflammatory responses. Atherogenesis is far from being completely understood, and here we summarize the main pathological factors contributing to the development of this disease, focusing on the role of lipids.

Atherosclerosis is a disease of the intima of the arteries, leading to pathological thickening of the arterial wall with other secondary negative effects on the health of patients. The problem with this disease is that the exact causes of atherosclerosis are still not clarified, which limits the development of effective anti-atherosclerotic therapy. Thus, the identification of key molecular changes happening during the initiation and further progression of atherosclerosis, including the understanding of the role of lipids in this process, is a task of vital importance.

Atherosclerosis is an inflammatory disease associated with the infiltration of immune cells into the arterial wall (1, 2). Atherogenic factors (such as desialylated, oxidized, and electronegative LDL) (3, 4) are thought to be one of the main reasons for the accumulation of lipids in cells of the intima and macrophages, leading to the formation of so-called foam cells and further progression of atherosclerosis. There are gender differences in the development of atherosclerosis, including differences in lipid profiles (5).

Mitochondrial DNA mutations have been shown to be associated with atherosclerotic lesions, leading to the idea of a possible role of these mutations in the development of atherosclerosis (6). It should be noted that mitochondrial DNA mutations and mitochondrial dysfunction are associated not only with atherosclerosis (6), but also with a plethora of different diseases: non-alcoholic fatty liver disease, diabetes, polycystic ovarian syndrome, cancer, and various neurological diseases (7–9).

The components involved in the regulation of cholesterol metabolism (such as LDLR and PCSK9) can be considered potential targets for the development of drugs for the prevention of LDL-induced accumulation of cholesterol in cells (10, 11). It should also be noted also that PCSK9 may be associated with mitochondrial dysfunction (12), and even cancer in some cases (13).

In addition, atherosclerosis can be considered an autoimmune disease (14), and thus certain approaches used for the treatment of autoimmune and autoinflammatory diseases (15–17) may be potentially applicable to the cure of atherosclerosis.

Evidence is accumulating that non-coding RNAs are involved in the development of cardiovascular diseases including atherosclerosis (1), and, in addition to that, exosomes may also be involved in the progression of this disease (18).

**Figure 1:**
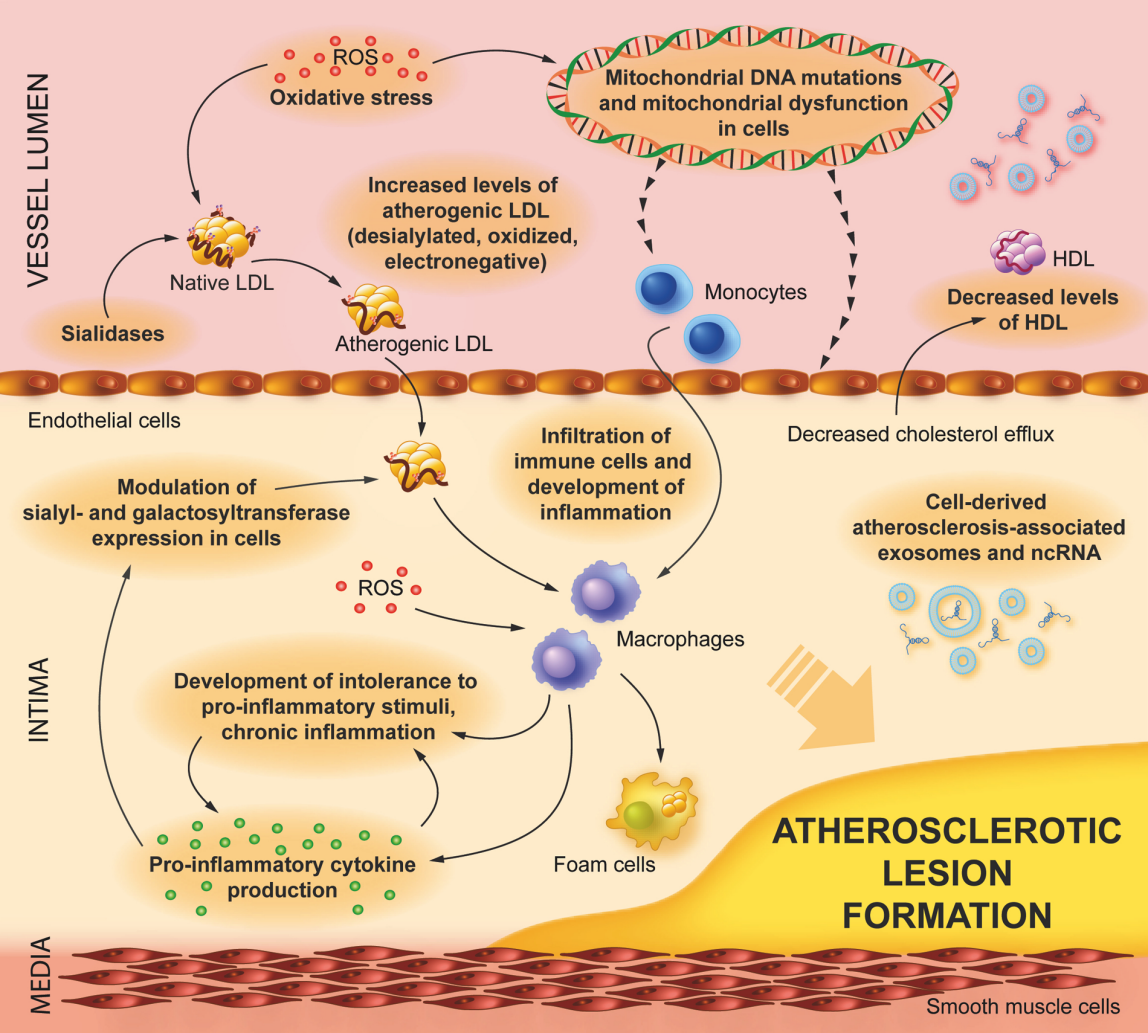
Pathological factors contributing to the development of atherosclerosis. Atherogenic LDL (desialylated, oxidized, and electronegative ones) induces lipid accumulation in endothelial cells and infiltrating macrophages, leading to foam cell formation, inflammation, and further progression of atherosclerosis. There are additional factors that contribute (or potentially contribute) to the development of atherogenesis, such as low levels of HDL, sialidase, and sialyl/galactosyltransferase activities leading to the generation of atherogenic LDL, intolerance to pro-inflammatory stimulation (including stimulation by atherogenic LDL), atherosclerosis-associated non-coding RNA (ncRNA), and exosomes.

Current pathological factors contributing to the development of atherosclerosis are shown in Figure 1.

This Research Topic collects 10 articles and reviews that are briefly discussed below.

A review by Poznyak et al. summarizes all known data on hypertension as a risk factor for atherosclerosis and risk assessment. The role of lipids in hypertension is discussed.

Data on oxidative stress as a primary factor linking lipids, inflammation, and atherosclerosis, in addition to therapeutic approaches to reduce oxidative stress were reviewed by Bale et al.

The reduction of plasma and liver lipid levels and atherosclerosis was discovered by Padalkar et al. in combined adenine-induced chronic kidney disease and diet-induced atherosclerosis in mice with a familial hypercholesterolemia mutation in the *ldlr* gene, leading to the conclusion that excessive adenine affects lipid metabolism.

The association of monomeric C-reactive protein (which is produced at sites of local inflammation) with carotid plaque number was found in patients with subclinical atherosclerosis in the study done by Melnikov et al.

The effects of lipoproteins, membranes, and extracellular vesicles on the structure and function of C-reactive protein were reviewed by Potempa et al.

Recent advances and future directions of research on the Von Willebrand factor (which may contribute to inflammation in atherosclerosis) with a focus on the diagnosis and treatment of cardiovascular diseases were reviewed by Kozlov et al.

The effect of Bie-Jia-Ruan-Mai-Tang (BJ, a traditional Chinese medicine formula) on proliferative diabetic retinopathy (the disease closely associated with inflammation) was investigated by Liu et al. using *in vitro* [human retinal capillary endothelial cells (HRCECs)] and *in vivo* (a diabetic mouse model) experiments.

The effects of forsythiasides [phenylethanol glycosides from the plant *Forsythia suspensa (Thunb.) Vahl*] on cardiovascular protection, anti-inflammation, antioxidation, and neuroprotection were reviewed by Yang et al.

The mechanism of action of monocyte locomotion inhibitory factor (MLIF) related to the reduction of ischemic stroke-induced inflammatory injury was investigated by Lv et al. with the conclusion about the anti-inflammatory effect of MLIF through the suppression of JNK/AP-1.

The attempt to identify molecular mechanisms of osteogenic differentiation of human valve interstitial cells (VIC) isolated from healthy donors or patients with calcific aortic valve disease associated with inflammation by RNA-seq transcriptomics and by shotgun proteomics was conducted by Semenova et al. with the finding of pro-osteogenic role of upregulated ZBTB16 gene.

The editors of Research Topic hope that the high standard of publications established in the first two volumes will be maintained in the third one.
